# Detection and Characterization of a Novel Norovirus in Bats, China

**DOI:** 10.1007/s12250-018-0010-9

**Published:** 2018-03-05

**Authors:** Ling’en Yang, Quanxi Wang, Lin Xu, Changchun Tu, Xiaohong Huang, Biao He

**Affiliations:** 1 College of Animal Science, Fujian A & F University, Fuzhou, 350002 China; 2 Key Laboratory of Jilin Province for Zoonosis Prevention and Control, Military Veterinary Institute, Academy of Military Medical Sciences, Changchun, 130122 China; 3 Jiangsu Co-innovation Center for Prevention and Control of Important Animal Infectious Diseases and Zoonosis, Yangzhou, 225009 China

Dear Editor,

Noroviruses (NoVs) are second only to the rotaviruses as etiologic agents of acute fulminant gastroenteritis in infants and young children worldwide, with an estimated 200,000 deaths per year in children younger than 5 years of age in developing countries (Patel et al. 2008). NoVs are classified within the genus *Norovirus* of the family *Caliciviridae* with Norwalk virus as its prototype member (ICTV 2017). The virions are small (38–40 nm in diameter) nonenveloped, with an icosahedral capsidanda linear, positive-sense, single-stranded, 7.7 kb RNA genome that is organized into 3 open-reading frames (ORF) (Green 2013). While only one species is currently recognized within the genus, molecular epidemiological studies have demonstrated a marked genetic diversity among circulating NoVs, and genotyping has been proposed based on relatedness within the complete VP1 capsid protein (Green 2013). Such a system has divided members into 6 major phylogenetic clades or genogroups, designated GI through GVI and sharing 46.1%–58.8% nucleotide (nt) identities in their *VP1* gene. Clades GI, II, and III are further subdivided into 9, 21, and 3 genotypes, respectively, indicating the need for further characterization of species criteria (Green 2013).

NoVs have been detected in a number of mammalian species (De Graaf et al. 2017). Strains infecting humans are found in GI, GII and GIV, while porcine NoVs group in distinct genotypes within GII, bovine and ovine viruses belong exclusively to GIII, murine NoVs are grouped in GV, canine NoVs are in GIV and GVI, and lion viruses have been found only in GIV (Green 2013).

As one of the most widely distributed mammals, bats are important natural reservoirs of viruses, of which more than 137 have been discovered (Luis et al. 2013), including many highly pathogenic agents such as Hendra and Nipah viruses (Yob et al. 2001), SARS-related coronaviruses (Ge et al. 2013), and Marburg virus (Amman et al. 2012; Towner et al. 2009). Bats have also been found to harbor diverse caliciviruses, in countries including China, Hungary, and Cameroon, all showing close relationship with members of the genus *Sapovirus* (Kemenesi et al. 2016; Tse et al. 2012), Nevertheless, only one sequence (accession number: KJ790198) and several 660 nucleotides (nt) amplicons of bat-borne NoVs, from China, has been reported (Hu et al. 2017).

**Fig. 1:**
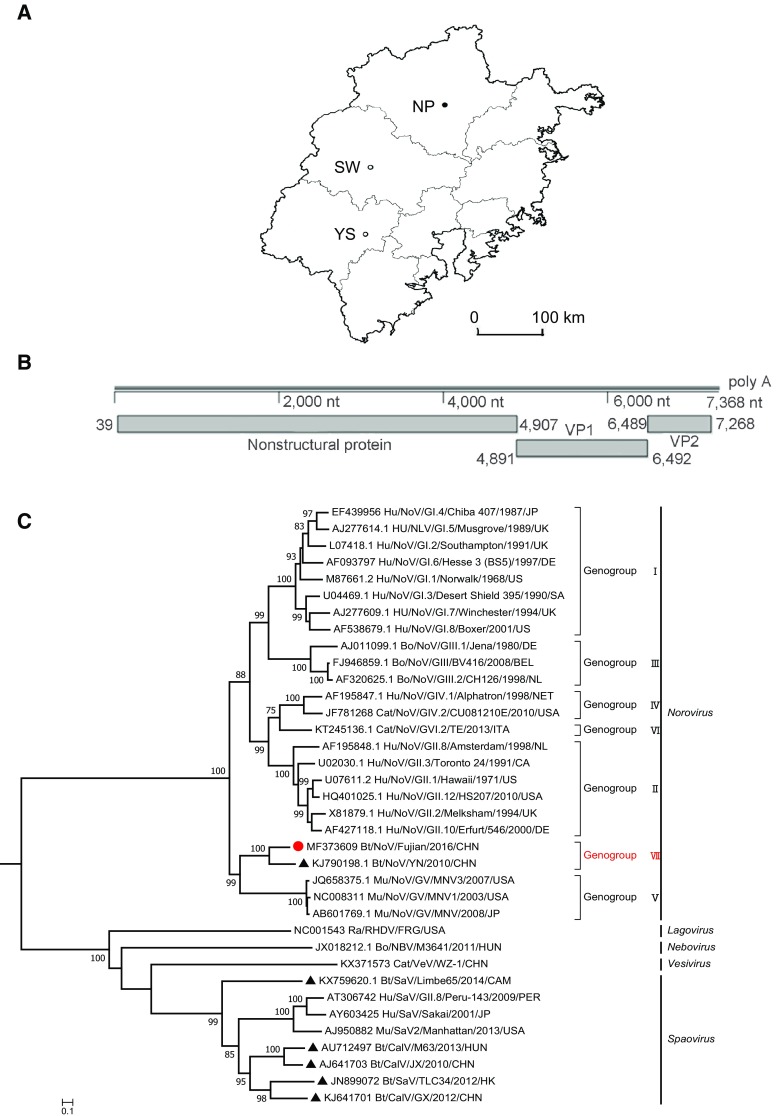
**A** Sampling location of this study, filled circles: bats positive for norovirus; open circles: negative. **B** Genomic structural schematic representation of NPIH26. Boxes represent the open reading frames encoding main proteins, nonstructural protein (39–4907 nt), VP1 (4891–6492 nt) and VP2 (6489–7268 nt). **C** Phylogenetic analysis of NPIH26 (filled red circle) and representatives of other caliciviruses based on their complete VP1 amino acid sequences. Other bat-borne caliciviruses are indicated by filled black triangles. Genogroups within the genus *Norovirus* are shown next to brackets, with the new genotype proposed in this study in red.

In 2016, 178 adult bats were collected from Yanshi, Shawu and Nanping in Fujian province, southeast China. The collection comprised 4 species: *Rhinolophus sinicus* (Yanshi, n = 48; Nanping, n = 1), *Hipposideros armiger* (Nanping, n = 10), *Rhinolophus affinis* (Nanping, n = 62; Shawu, n = 21), and *Myotis horsfieldii* (Shawu, n = 36) (Fig. 1A). All bats were live-captured with nets near or within human inhabited communities, and were apparently healthy at capture. Anal specimens were collected using sterile swabs, immediately transferred to viral transport medium, and stored in liquid nitrogen after sample collection, as described previously (He et al. 2017). As part of another investigation, all were tested for group A rotaviral RNA and found negative. Samples from each location were pooled and subjected to viral metagenomic analysis as per our published method (He et al. 2013).Ten contigs were annotated to NoVs, with an average length of 189 nt and showing 68%–79% nt sequence identity with other known NoVs, these were used to design PCR primers, targeting 239 nt of the polyprotein gene (forward primer sequence: 5′-CAACTGTGACCGCATAGAG-3′; reverse primer sequence: 5′-TGAGGACGAGGTGGGAATG-3′). RNA of the samples was extracted using QIAamp RNA Mini Kit (Qiagen, Hilden, Germany), and subjected to RT-PCR screening, with 35 circles of 94 °C for 30 s, 58 °C for 30 s and 72 °C for 30 s. RT-PCR with double-distilled water replacing the RNA was included as a negative control. Results showed that 2 of 62 (3.2%) *R. affinis* collected from Nanping were positive. Preliminary phylogenetic inspection of the amplicons showed that they shared 99.8% nt identity, and the viruses from which they originated were named NPIH26 and NPIH29. The genomic sequence of NPIH26 was then determined using a long-range PCR system (TaKaRa, Dalian, China) and Rapid Amplification of cDNA Ends (RACE, TaKaRa). Results showed that the complete genome (GenBank accession number: MF373609) was 7368 nt in length with 58.05% CG content and possessed genomic organization typical of noroviruses, containing three ORFs encoding the multifunctional nonstructural protein, VP1 and VP2, as well as 5′ leader and 3′ trailer sequences (38 and 33 nt respectively) (Fig. 1B). The conserved motifs DYXX(TR)WDST, GLPSG and YGDD were observed and the genome contained the same structures in the RNA-dependent RNA polymerase region as other NoVs (Kemenesi et al. 2016; Smiley et al. 2002; Wolf et al. 2011).

Genomic comparison with representatives of the six NoV genogroups and other caliciviruses showed that NPIH26 shared the highest nt identity (73%) with the bat NoV BtRs-CalV/YN2010 identified in *R. sinicus* from Yunnan, China (Genbank accession number: KJ790198), although lacking ~ 1000 nt at the 5′ end. Shared identities with other NoVs, were ≤ 59.6% and ≤ 46.7% with other caliciviruses including bat caliciviruses (Table 1). To determine the phylogenetic relationship, the *VP1* genes of these viruses were aligned using ClustalW, available in MEGA6, and a phylogenetic tree was constructed by the maximum likelihood and general time reversible models under evaluation of 1000 bootstraps. The viruses clustered within five groups corresponding to their genera within the family *Caliciviridae*, with NPIH26 placed with other NoVs but forming a phylogenetic group with BtRs-CalV/YN2010 separate from the six currently known genogroups. Accordingly, these bat-borne NoVs can be considered as comprising a new genogroup, GVII (Fig. 1C). ORF1 and VP2 were also used for phylogenetic analyses and showed similar topology with that of VP1 (data not shown).

**Table 1:** Amino acid and nucleotide sequence identity comparison between NPIH26 and other representatives

Strains	Genbank accession #	Full sequence		RdRp				VP1				VP2			
		nt	%	nt	%	aa	%	nt	%	aa	%	nt	%	aa	%
NPIH26		7368		4869		1622		1602		533		780		259	
BtRs-CalV/YN2010	KJ790198	6582	73.0	3999	70.4	1332	78.0	1623	61.3	540	54.5	753	63.9	250	56.5
Human norovirusGI	MB7661	7654	54.4	5370	55.3	1789	52.0	1593	43.3	530	45.9	639	28.2	212	26.5
Pig norovirusGII	HQ392821	7548	59.6	5082	61.3	1693	62.2	1644	61.3	547	43.6	762	44	253	39.0
Bovine norovirusGIII	AJ011099	7338	57.8	5043	56.4	1680	53.7	1560	53.9	519	46.1	672	33.5	223	21.4
Human norovirusGIV	NC029647	7527	59.0	5064	59.9	1687	60.4	1671	59.1	556	40.3	729	40.8	242	37.0
Murine norovirusGV	AY228235	7382	57.2	5064	54.8	1687	49.6	1626	57.6	541	48.0	627	33.9	208	28.7
Dog norovirusGVI	FJ692501	7637	58.0	5076	60.5	1691	61.1	1587	65.0	528	49.0	840	41.9	279	37.7
Bat sapovirus	NC033776	7475	41.7	5160	32.2	1720	17.6	1659	28.6	552	18.6	492	17.4	163	13.1
Porcine sapovirus	AF182760	7320	42.3	5130	32.4	1710	18.1	1635	29.5	544	17.2	495	18.9	164	8.9
Human sapovirus	AJ249939	7490	42.5	5160	27.4	1720	18.9	1683	27.9	560	14.7	495	20.8	164	12.8
Lagovirus	M67473	7437	46.7	5290	33.0	1763	17.7	1745	29.3	581	12.6	354	14.2	117	4.4
Nebovirus	DQ013304	7454	42.1	4702	31.7	1567	18.1	1931	29.3	643	10.2	678	26.1	225	11.6
Vesivirus	U76874	8284	33.6	5646	30.5	1881	18.6	2106	27.0	701	15.1	333	12.0	110	6.0

nt, nt length; %, identity; aa, aa length; BtRs-CalV/YN2010 lacks ~ 1000 nt at 5′ end

Kunming mice (3 day-old) were inoculated intraorally with positive sample NPIH26 for virus isolation with daily inspection for any visible pathology. At 7 days, without showing any signs of morbidity or weight loss, all mice were euthanized and their organs (intestines with contents, lungs, livers, kidneys and spleens) were removed and subjected to specific RT-PCR detection. All organs tested negative for this virus.

Of all the noroviruses, only murine strains have been propagated in cell culture. Consequently, the inability to cultivate human NoVs in vitro has been a major obstacle to the development of antivirals and vaccines to cure or prevent human noroviral diseases (Green 2013). A recent breakthrough study, however, has shown that human NoVs can replicate efficiently in stem cell-derived multicellular human intestinal epithelial cultures called enteroids, which may overcome this obstacle (Ettayebi et al. 2016).

Noroviral infections of children in China and the rest of the world pose a huge health and financial burden on their families and the communities (Ahmed et al. 2014). No case of human noroviral diarrhea has so far been found to have an animal origin, as viruses of different animal origin have evolved within distinct genogroups (Fig. 1C). However, phylogenetic analyses have shown that some human NoVs (e.g. Hu/NoV/GIV.1/Alphatron/1998/NET, Fig. 1C) share a close relationship with non-human viral members of the genus (e.g. Cat/NoV/GIV.2/CU081210E/2010/USA, Fig. 1C), which illustrates the complicated evolutionary history of NoVs of different host origins. Bats can spread many pathogens to humans, including those causing diarrhea; e.g. group A rotaviruses (He et al. 2017). So far only a few NoVs have been reported in bats, but there is a high likelihood that more strains are circulating in the bat populations. The divergent relationships of bat NoVs with other NoVs may help our understanding of evolutionary origins of this virus.
